# Hospital infection and COVID-19: Do not put all your eggs on the “swab” tests

**DOI:** 10.1017/ice.2020.254

**Published:** 2020-05-27

**Authors:** Francesco Chirico, Gabriella Nucera, Nicola Magnavita

**Affiliations:** 1 Postgraduate School of Occupational Medicine, Università Cattolica del Sacro Cuore, Rome, Italy; 2 Health Service Department, State Police, Ministry of Interior, Milan, Italy; 3 Faculty of Nursing, University of Milan, Italy; 4 Fatebenefratelli Sacco, FatebeneFratelli Hospital, Milan, Italy; 5 Postgraduate School of Occupational Medicine, Università Cattolica del Sacro Cuore, Rome, Italy; 6 Department of Woman/Child and Public Health, Fondazione Policlinico “A.Gemelli” IRCCS, Rome, Italy

*To the Editor*—In healthcare settings, including long-term care facilities, hospital administrators have a legal obligation to set up a risk assessment strategy to carry out effective prevention and control measures during the management of suspected and confirmed cases of COVID-19 infection.^1^ Hospitalized inpatients and residents in care homes are often elderly and immune-depressed patients with comorbidities; thus, they are at high risk of infection and mortality. Special attention and efforts to protect or reduce transmission should be also applied in healthcare providers because depletion of the healthcare workforce not only will affect health care but also will contribute to the spread of the outside hospitals.

According to European Centres for Disease Control and Prevention (EU-CDC) guidelines,^1^ each hospital should constitute a ‘COVID-19 preparedness and response committee’ and should prepare a specific plan, including a number of administrative and structural measures for patient and healthcare management. Undoubtedly, the most important measure in reding the likelihood of nosocomial infection is early isolation of patients with COVID-19, or at least maintaining a safe distance between those who are awaiting diagnosis. However, a number of recent studies showed that patients with mild or nonspecific symptoms can escape isolation and thus introduce SARS-CoV-2 into hospitals, leading to clusters of nosocomial infections.^2^

To minimize the risk of spreading, mass testing with nasopharyngeal and oropharyngeal (NP/OP) swab of all patients has been proposed,^2,3^ associated with mass testing of both symptomatic and asymptomatic healthcare workers.^4^ Even the use of these expensive and demanding mass strategies, however, cannot be considered a measure of absolute guarantee.

Indeed, Xie et al^5^ observed typical COVID-19 chest lesions via computed tomography (CT) scans in 5 patients with a negative or weakly positive swab test (RT-PCR test). Another patient with a chest X-ray showing interstitial pneumonia but with a negative RT-PCR test was reported by Winichakoon et al.^6^ Kumar et al^7^ reported the case of a patient with pneumonia and negative nasopharingeal swab who tested positive some days later with a bronchial lavage sample. Bandirali et al^8^ found that asymptomatic or minimally symptomatic patients may have abnormalities in chest x-rays after 14 days of quarantine, with a sensitivity of RT-PCR testing of 59%.^8^

Cao et al^9^ observed that patients with negative to RT-PCR tests may have specific IgG and/or IgM for SARS-CoV-2 at recovery stage.^9^ In reality, the sensitivity of an NP/OP swab in the course of disease ranges between 42% and 71%^6^ and depends on sampling technique, timing within the clinical course of COVID-19, and viral loads detected in the swab.^6^

In conclusion, given the fact that negative NP/OP swabs do not rule out COVID-19 diagnosis, we propose that all the patients hospitalized with pneumonia be subjected to swab obtained by deep tracheal aspirate, which has a lower risk of aerosolization.^10^ We further recommend that suspected infection be checked with a combination of repeated RT-qPCR tests and chest CT scan. All patients hospitalized without respiratory symptoms should also be checked with repeated RT-qPCR tests and chest X ray before admission in hospital wards.

Moreover, healthcare providers should be tested regularly with serological test and swabs and symptom monitoring. Finally, a policy of universal masking and eye shielding for all healthcare providers involved in direct patient care is needed.
